# Participatory Design of an Electronic Medical Record for Paediatric Palliative Care: A Think-Aloud Study with Nurses and Physicians

**DOI:** 10.3390/children8080695

**Published:** 2021-08-12

**Authors:** Sven Kernebeck, Theresa Sophie Busse, Chantal Jux, Dorothee Meyer, Larissa Alice Dreier, Daniel Zenz, Boris Zernikow, Jan Peter Ehlers

**Affiliations:** 1 Chair of Didactics and Educational Research in Health Science, Faculty of Health, Witten/Herdecke University, 58448 Witten, Germany; theresa.busse@uni-wh.de (T.S.B.); chantal.jux@uni-wh.de (C.J.); jan.ehlers@uni-wh.de (J.P.E.); 2 PedScience Research Institute, 45711 Datteln, Germany; d.meyer@pedscience.de (D.M.); l.dreier@pedscience.de (L.A.D.); b.zernikow@kinderklinik-datteln.de (B.Z.); 3 Department of Children’s Pain Therapy and Pediatric Palliative Care, Faculty of Health, School of Medicine, Witten/Herdecke University, 58448 Witten, Germany; 4 Smart-Q Softwaresysteme GmbH, Lise-Meitner-Allee 4, 44801 Bochum, Germany; zenz@smart-q.de; 5 Pediatric Palliative Care Centre, Children’s and Adolescents’ Hospital, 45711 Datteln, Germany

**Keywords:** palliative care, paediatrics, participatory design, electronic health records, electronic medical records, technology acceptance, usability, user involvement

## Abstract

Background: Electronic medical records (EMRs) offer a promising approach to mapping and documenting the complex information gathered in paediatric palliative care (PPC). However, if they are not well developed, poorly implemented EMRs have unintended consequences that may cause harm to patients. One approach to preventing such harm is the involvement of users in the participatory design to ensure user acceptance and patient safety. Therefore, the aim of this study is to evaluate the acceptance of a novel patient chart module (PCM) as part of an EMR from the perspective of potential users in PPC and to involve these professionals in the design process. Methods: A qualitative observational study with N = 16 PPC professionals (*n* = 10 nurses, *n* = 6 physicians) was conducted, including concurrent think aloud (CTA) and semi-structured interviews. A structured content analysis based on the Unified Theory of Acceptance and Use of Technology was applied. Results: The results can be summarized in terms of general observations, performance expectancy, effort expectancy and facilitating conditions, all of which are likely to have a positive influence on acceptance of the PCM from the user perspective in the context of PPC. Conclusions: The involvement of users in the development of EMRs is important for meeting the requirements in PPC. Further software adaptations are necessary to implement these requirements.

## 1. Introduction

Paediatric palliative care (PPC) encompasses a holistic care approach for children and young adults with life-threatening and life-limiting diseases and their families [1,2]. Neurological, genetic and metabolic diseases, rather than malignancies, are the most common diagnoses [3,4]. Major goals of PPC include management of symptom relief and maintaining the highest possible quality of life of patients and families by providing ongoing support to affected families using a multidisciplinary care approach [5,6].

In different aspects, patients undergoing PPC fundamentally differentiate from adult palliative patients and even from paediatric patients in general: children are often born with very rare life-limiting conditions, whereas adult patients develop diseases in the later course of life. Over the course of the disease of patients undergoing PPC, care and treatment in different settings are necessary, resulting in many sector changes and corresponding high clinical documentation demands [7,8]. Even within a facility, numerous professionals whose activities must be carefully documented and coordinated are involved in a child’s care process [9]. Moreover, the processes of prescribing medications differ significantly between children and adults. For example, prescribing medications in the care of children is complex because the dose for a variety of medications varies by age, body surface area [10] and the need for off-label medication [11]. This makes the professionals particularly vulnerable to medical errors [12].

Electronic medical records (EMRs) offer a promising approach to mapping and documenting the complex information gathered in PPCs [13]. EMRs are digitized medical charts for use in clinical practice by healthcare professionals within an organization [14]. With EMRs, administrative and health-related information can be captured, stored, transmitted, and displayed from different sources [15]. They incorporate comprehensive health-related information, such as medical history, medication orders, vital signs, or laboratory results [16,17]. The use of EMRs has been associated with improving treatment quality and patient safety [16], reducing the workload for health care professionals, reducing costs of healthcare [18], and enhancing the coordination of care [19]. These advances can be achieved through the integration of electronic decision support systems or predefined clinical standards and guidelines to access the latest medical knowledge [16,20].

However, if they are not well developed, poorly implemented EMRs can have several unintended consequences [21,22]: large and unstructured data sets create a high cognitive demand for users to locate, contextualize, and summarize information [23]. In addition, they may create unintended workflow disruptions, causing cognitive and physical burdens because of the management of such workflows [24,25]. The resulting dissatisfaction with EMR use impacts healthcare professionals such as nurses and physicians, potentially leading to burnout, fatigue or stress and time pressure [26,27,28]. EMRs can also be a source of adverse drug events related to improper prescription of drugs that can jeopardize patient safety [21,29]. Unintended consequences may also stem from EMRs that are not sufficiently well aligned with users’ needs [21] and, most importantly, have poor usability [25].

Therefore, the specific needs of users, the context and requirements of the clinical environment and the related sociotechnical interactions has to take into account in the development of EMRs to avoid these unintended consequences [21,30]. For this purpose participatory design by the involving (future) users as “experts by experience” is considered as a main approach [30,31]. Participatory design focuses on involving users in a creative development process, respecting four main principles [31]: (1) democratization of decision-making processes, (2) mutual learning processes, (3) observation of latent (implicit) knowledge structures and (4) mutual creativity through collaborative work between developers, researchers and (future) users.

In participatory design, user acceptance plays a central role [31,32]. The theoretical basis is provided by acceptance models such as the “Unified Theory of Acceptance and Use of Technology (UTAUT; [33,34])”. The UTAUT is considered as one of the main frameworks for the evaluation of EMR [35]. The UTAUT incorporates four core direct determinants of the behavioural intention to use a technology from a user perspective: performance expectancy, effort expectancy, social influence and facilitating conditions. Performance expectancy is defined as “The degree to which an individual believes that using the system will help him or her to attain gains in job performance” [34] and encompasses mainly the functionalities of a technology. Effort expectancy is defined as “The degree of ease associated with the use of the system” [34], which basically includes the dimension of the perceived usability and complexity of use. Social influence is defined as “The degree to which an individual perceives that important others believe he or she should use the new system”. Facilitating conditions are defined as “The degree to which an individual believes that an organizational and technical infrastructure exists to support use of the system”. To take the needs of the users into account, these determinants must be addressed in the participatory development of new technologies.

To the best of our knowledge, no EMR specifically for the setting of PPC has been developed involving (future) users in a participatory design process. In PPC, the situation today is that documentation is often performed with EMRs that were developed for adult patients in palliative care. In general, EMRs are inherently complex systems with a large number of different interacting components. Each of these components requires special attention in participatory development and evaluation.

Therefore, the aim of the study is to evaluate the acceptance of a novel patient chart module (PCM) that has been prototypically developed as part of an EMR for PPC from the perspective of potential users.

This study is based on the following research question:

How do potential future users in PPC perceive a novel PCM in terms of technology acceptance, and what are wishes for improvement?

## 2. Materials and Methods

### 2.1. Research Design

A qualitative observational study was conducted in which each session included the concurrent think aloud (CTA) method, followed by a semi-structured qualitative interview.

In CTAs, participants are asked to verbalize their feelings and emotions directly while interacting with a (new) technology [36,37]. Through this, the “black box” of emotional and cognitive processes as well as reactions can be observed and evaluated; recall problems can potentially be minimized [38,39]. Since the early 1990s, CTA has been used in human–computer interaction, and today, it is also used in the participatory design of technologies in health care [31,39]. From user-based methods, CTA is considered one of the “gold standards” [40].

Ethical approval was obtained by the ethics committee of Witten/Herdecke University (approval code: 35/2019).

### 2.2. Participants and Recruitment

PPC professionals working in a PPC unit of a children’s hospital in Germany were recruited as potential participants for this study. For this, posters and flyers were distributed to inform them about the study’s aim and procedure. In addition, professionals were personally contacted by members of the study team. If the potential participants expressed interest in taking part in the study, verbal and written information material, as well as an informed consent form, was handed out.

The inclusion criteria were as follows: (1) person actively working as a PPC physician or nurse and (2) capacity to give informed consent prior to the study. No particular knowledge in the application of EMRs or other health-related technologies was necessary.

All participants gave their informed consent to participate in the study. An expense allowance of 40 € per hour was paid to all participants.

### 2.3. Patient Chart Module (PCM)

**Figure 1:**
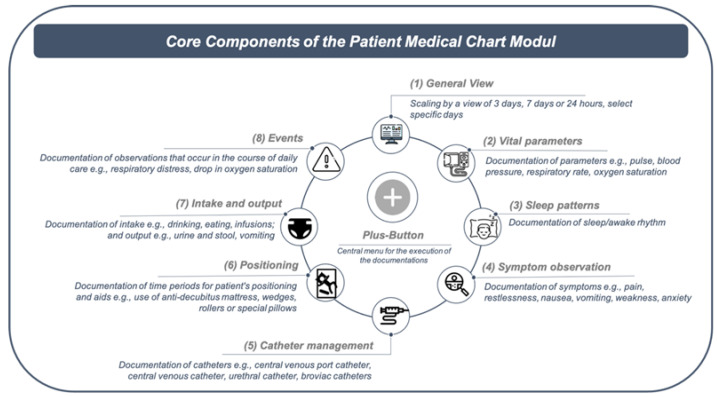
Overview of the core components of the patient chart modul. (Icons made by Freepik from www.flaticon.com, accessed on 28 June 2021).

The novel PCM to be evaluated in this study was created within the framework of the electronic cross-sectoral health record for paediatric palliative care (ELSA-PP) project, which aims to develop an EMR specific for the context of PPC (Figure 1). During the project, an EMR originally developed for adult palliative care (ISPC, company: smart-Q, Germany) is successively adapted to the setting of PPC. The software (ISPC) into which the PCM is integrated has been used for many years for adult patients in palliative care. The software was developed by engineers and software developers, with feedback from users incorporated into the adaptation over the years. However, the PCM module is a fundamentally new module, which was newly developed within the framework of the ELSA PP project. For PCM development, the needs of PPC professionals (e.g., nurses, physicians) towards such a technology were surveyed in profession-specific focus group interviews and one-on-one interviews prior to this study [13].

As a result of the needs assessment, the PCM was developed and can be characterized as an early prototype [41]. The main intended user groups are nurses and physicians. Based on clinical tasks that nurses and physicians perform in clinical practice, the PCM comprises eight core components (Figure 1), for which information can be documented via a central menu called the “plus-button” (Appendix A). When the plus button is clicked on, an overview of the PCM core components appears first, which can then be selected accordingly to document the relevant information. Since the plus-button is visible and selectable on every view of the EMR, this is meant to give users quick access to the components that are relevant to them at any given time.

### 2.4. Study Procedure

To evaluate the PCM, participants were asked to complete standardized tasks in the eight core components that were representative of everyday clinical practice (e.g., documenting vital signs; [40,42]) and presented as a printed-out leaflet (Appendix B).

All sessions took place in a standardized setting in a regular office with the same software and hardware setup. The hardware setup included a desktop computer and a monitor mirrored with it for the study team researchers to observe the participants’ actions. Screen movements and audio were recorded via screencast. During each session, two researchers were present, with one conducting the session and one taking notes. The PCM was filled with dummy data for the sake of providing a scenario that was as descriptive as possible [43].

At the beginning of a session, participants were informed verbally about the CTA procedure. They were encouraged to read aloud each task first and then to continuously verbalize their thoughts while performing these tasks. If necessary, the researchers reminded them to verbalize their thoughts. This reminder is recommended, as it is known that participants often stop verbalizing their thoughts during CTA [44]. To create an atmosphere of comfort, it was pointed out that the CTA should not be about the “right or wrong” performance of the tasks, but rather that the participants’ personal opinions about the PCM were to be assessed [43]. To analyse the learning ability and the self-explanatory capability of the PCM, only a short overview of its central functions and the plus button was explained. During the execution of the tasks, interactions with participants were kept to a minimum [36]. When questions or problems arose during testing, specific contextual responses or queries were made by the lead researcher. Basically, the approach chosen here is open-ended and exploratory to give users the opportunity to verbalize their thoughts and to discuss with them afterwards, as is recommended in such user-based evaluations [43].

After the CTA, a qualitative interview was conducted using an interview guide (Appendix C) created in advance [45]. Participants were asked about their general impressions, needs regarding content and functionalities, structure, design and usability of the PCMs and what wishes they would recommend in terms of improvement, for example. Furthermore, participants were asked how they felt about the study situation and procedure during the CTA.

### 2.5. Data Analysis

Audio files were transcribed verbatim, anonymized, and transferred with the video files into MAXQDA 2020 software. Two researchers independently performed qualitative structured content analysis (S.K., T.S.B.) within an iterative process [46]. Statements and observations were mapped deductively to the four main determinants of UTAUT as the main categories. Subsequently, inductive subcategories were grouped within these categories and assigned to the eight PCM core components [47,48]. As a result, a comprehensive coding framework evolved. Disagreements in coding were resolved through discussions and consensus building (S.K., T.S.B.). To ensure intersubjective comprehensibility, codings were then discussed with two other researchers of the study team (L.A.D., D.M.). Participants’ quotes were translated into English for this article. In addition, pseudonyms were assigned in the following manner: (profession_ transcript-ID_#timecode#).

## 3. Results

In total, CTAs with *N* = 16 PPC professionals (*n* = 10 nurses, *n* = 6 physicians) were conducted (mean duration: 43 min, range: 32–86 min). Of the participants, *n* = 11 were female, and *n* = 5 were male. An overview of the participants’ characteristics is provided in Table 1.

Overall, the analysis resulted in four main categories and 42 subcategories (Table 2): (1) general observations, (2) performance expectancies, (3) effort expectancies, and (4) facilitating. No observations or statements could be grouped regarding social influence. The subcategories of performance expectancies and effort expectancies were summarized grouped to the components of the core PCM (Figure 1) in the reporting of the results. Due to the large number of observations and statements, only serious or frequently mentioned contents are reported herein.

### 3.1. General Observations

The majority of the participants experienced the PCM to be intuitive in application and self-explanatory. Familiarization with the PCM increased over the time of application.

*“Well, of course it’s a bit strange at first to do everything online on a PC, because you’re just used to writing. And I also thought at first that it would be totally difficult because I don’t really get along with computers, but I found it relatively simple and easy to understand. So, I often didn’t have to think long about where to find it and so on. It was already clear. I thought that was good in any case. I would have imagined it to be more difficult.”*

*(Nurse_08_#00: 35:48#)*

In addition, the presentation of information in the PCM was perceived as clear and concise. Here, it was felt to be positive that a quick overview of the patient’s status is available at a glance. Electronic documentation in general was reflected as having a number of advantages compared to analogue documentation, for instance, regarding the potential increase in clarity and the fact that relevant information is stored in one place.

*“I find them very clear. So, because with the PCM everything can be seen relatively quickly. I don’t have to go through umpteen sheets. I think that’s totally good. Yes. Otherwise. Yes, that you can jump back and forth very quickly. […] You have a quick overview of what you want to search for. I find the buttons so, so the individual sub-items listed quite well, so that you can also find it well and quickly. Exactly.”*

*(Nurse_04_#00:44:15#)*

A proportion of participants found the PCM to be overwhelming at first glance due to the unfamiliarity and amount of information being provided. It was emphasized several times that a high level of familiarity with the PCM is necessary. In addition, it was perceived as useful to have only those elements appear in the PCM that are relevant to the patient’s situation.

*“First of all, a lot of information for the beginning, a lot in the overview. Where you first have to get used to it, just get a bit of an overview. After a little bit of use, it was quite clear, I must say. I think it’s good that […], that not all menus appear here immediately. Because I think otherwise it’s going to overwhelm you. If now in and out with a patient is actually no problem at all, we also have patients who have no [Catheter] or anything similar. Then it’s not in there either. I think it’s good that you don’t have to worry about it and that it only appears when I really actively enter something in it. I also like the fact that you can do all this via this menu [Plus Button] item here and then really select the individual things. I think that’s really good. Here with the curve, with the fever curve, I think you have to get used to it a little bit first.”*

*(Nurse_05_#00:49:49#)*

### 3.2. General View and Plus-Button

In the PCM general view, it was requested that a finer scaling of this view is necessary to view information in more detail (e.g., twelve and four hours).

Participants expressed considerable problems while switching between the views of one, three or seven days. One of the reasons for this was that the system required users who wanted to, for instance, switch from the three-day view to the seven-day view to re-enter the starting date each time. The participants described this process to require an unnecessary navigation action. Additionally, an even finer scaling, for example, by the hour, should be possible, as this is needed to meet the specific PPC demands.

*“[…] Yeah, okay I’ll get on the tasks here and look at one day, three days, seven days. That’s probably up here. And click on-. Oh, three days I have to apply. No. Oh, I have to select the days. Now I would have thought, for example, that it would automatically select the [...] the last three are.”*

*(Physician_15_#00:01:28#)*

*“Exactly, when many things are next to each other. Can you then scale, well there are scaling options at the moment, days, three days, seven days. And the question is, of course, whether the nursing staff might be happier, or the doctors, if you also have the option of eight hours, for example. So that one sees his shift there. Then it’s all bigger, easier to meet. […]. That will be at the latest when the medication process and so on is to be included. And they have a lot of medications.”*

*(Physician_13_#00:43:17#)*

**Figure 2:**
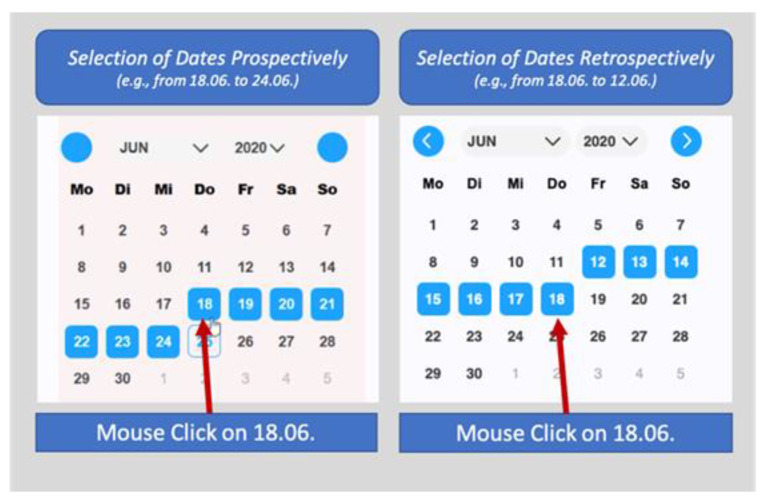
Prospective vs. retrospective selection of dates.

Participants also found it to be unintuitive that the selection of days had to be made prospectively instead of retrospectively. A visual explanation of this problem can be found in Figure 2.

*“Mhm, so I wanted seven days now, right? And of course, I choose the last seven days now first, that’s the most common, isn’t it? So, I would have expected that I now somehow have the 3rd of July here. Now I can’t get to June. So, I already have to set the-, the date up here. Like this, isn’t it? And I have already said, I want seven days, then of course it would make sense he would shaded back from three, give me the last seven days, so that I then-. Now I would have to count, one, two, three, four, five, six, seven, is the 25th, isn’t it?”*

*(Physician_13_#00:09:53#)*

**Figure 3:**
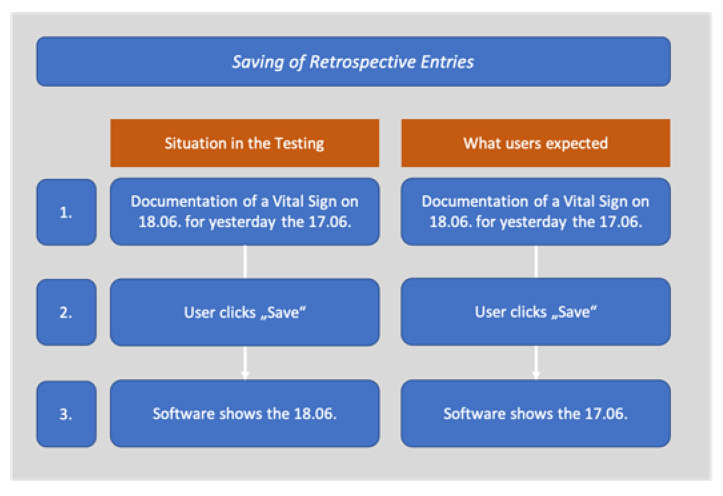
Retrospective saving of entries.

Regarding the plus-button, an often-observed problem concerned the saving of retrospective entries. For example, participants wanted to enter a vital sign for the day before while being in the PCM general view for the current day. However, after saving, the view did not switch to the day of the saved entries (the day before), which confused participants. Sometimes they accordingly had the impression that their entry was not saved. The wish here was that after saving entries retrospectively, the view would go directly to the date of entry (Figure 3).

*“I would have expected now, when I document on the 18th, that the 18th page also opens up [after saving]. And not the 17th […].”*

*(Nurse_02_#00:29:19)*

### 3.3. Vital Signs

In the documentation of vital signs, the documentation of a patient’s body weight caused three major problems according to participants. This related to (a) where body weight was located in the PCM, (b) how differentiated the body weight was depicted, and (c) how the updating of data entries worked.

It was perceived that displaying the patient’s body weight in a header above the vital signs was not appropriate, as the concern of overlooking this information arose (a). It was suggested that the weight information, for example, could be located directly below the vital signs.

The naming of the body weight in the header was perceived as not precise enough, as it did not indicate whether this was the patient’s last measured weight or whether it was the weight on admission to the PPC unit. As a solution, depicting both the weight at the time of admission and the current weight in the PCM was mentioned (b).

*“So, it has the advantage, if it is in the header, that it is immediately visible. The question is always: Is it then the current weight? Because the date of recording and the reason for recording are written here. So here you have the impression that there are, so to speak, the fixed quantities at time X. That this is now on the day weight and size, I would not expect now, if I consider the above as master data, first of all. Because that looks as if that’s something fixed that accompanies the patient over the whole time.”*

*(Physician_10_#00: 13:26#)*

A further wish commonly expressed was that the body weight data should be displayed graphically in some kind of curve to view and evaluate the corresponding changes (b).

*“With the weight, you could still maybe somehow-, that you have another line like that [the vital signs chart]. There is only the current weight. So, if I have now weighed today, that one has there somehow again what. Or a button that you can click on, so that you can see the weights. That you can see such a progression. Especially for children, where it is important. They come because they are really too skinny. Or they have to be weighed every day or something. I think that would be cool.”*

*(Nurse_02_#00: 45:32)*

After generating a new entry for the weight, it was observed that initially, this was not displayed, but that the page had to be refreshed first. This was found to be unnecessary and not intuitive to use (c).

Additionally, participants felt that it was sometimes difficult to distinguish which colour and which scale belonged to which vital sign. Additionally, the current display of the legend was perceived as confusing. This was because the labels of the vital signs overlapped in the presentation in the legend. It was important to the participants that the labels of the legend that are displayed for each of the different vital signs are more differentiated.

*“And now here with the vital signs is-, there are no labels now, right? So, the red one is just red and down here it says minutes or something, whatever that’s supposed to be. Because I don’t know what that is. It doesn’t say that anywhere, does it.”*

*(Physician_10_#00: 14:46#)*

### 3.4. Sleep Patterns

Even if the detailed depiction of a patient’s sleep or wakefulness was regarded as meaningful, the data entry was appraised as complicated and not intuitive. This was essentially related to the fact that only the state (e.g., sleep or wake) could be documented for a point in time (e.g., 7:00 a.m. patient asleep, 8:00 a.m. patient awake) rather than for a period of time (e.g., patient asleep from 7:00 a.m. to 8:00 a.m.). In addition, it was not possible to document only the beginning of a phase and add the end of it later, just as it was not possible to subsequently revise already saved entries. Both aspects should, however, be able to represent the clinical workflow as accurately as possible.

*“Exactly, the time span is the problem. That’s something, that’s unfortunate because that requires double steps. […] Document 12 to 12:30 bedside—will be the same. That means I have to do basically the same thing and I have to enter something twice. And then have to change the position again, so to speak. It’s doable, but it’s time consuming.”*

*(Physician_10_#00:28:55#)*

*“But exactly, I would find that cool too, if you can click on it and then just change it to sleep [from awake] if you did it wrong.”*

*(Physician_15_#00:20:10#)*

Regarding the localization of the sleep pattern block, participants felt it should be presented closer to the vital signs (e.g., directly below), allowing us to relate this information. This could be used, for example, to check whether a child shows seizures only at night and whether these are accompanied by drops in oxygen saturation.

*“[…] So that, I think, would be quite good if you could perhaps add that to the sleep/wake rhythm, that you could somehow mark a time of restlessness once again. […] Because we currently also do this with red lines and red bars when the child has a restlessness phase.”*

*(Nurse_05_#00:18:29#)*

In principle, this function was perceived as useful, although the level of detail in the display of seven days was not felt to be sufficient. It was described that when seven treatment days are presented, it is difficult to assess how sleep behaviour changes over time. The reason for this was that the presentation of the scaling of the sleep patterns line was too reduced in the view of seven days compared to the view of 24 h. When looking at seven days, the line of the sleep patterns was compressed in such a way that the changes over time could not be assessed.

*“But you don’t have a view of how the day and night rhythm changed during the week. That gets lost in the representation here. No. It’s just that I have a-, the same as our (paper-based) 24-h (sleep) protocol. But there you have it just among each other. And you see how a day and night rhythm shifts or forms. Evolves. And the info gets lost in here. That’s kind of difficult in the view, of course. We have this seven-day view now. But if I were to click on it now, make a double click, then maybe I would like to have the individual days one below the other.”*

*(Physician_09_#00: 45:12#)*

### 3.5. Symptom Observation

On the one hand, participants described that it was not necessary or possible to indicate the exact intensity (on a scale of 0–10) for all of the possible symptoms that PPC patients may exhibit. These symptoms should therefore be documentable without specifying intensity (e.g., light, medium, heavy), but the possibility of providing a comment. The possible value range in which the intensity of symptoms was to be specified should nevertheless be displayed (e.g., via mouse-over).

On the other hand, some of the symptoms, especially pain, should be captured by more sophisticated and standardized measures than a “simple” numerical scale with the results presented accordingly in the PCM.

*“There I go again on the plus and on symptoms. For example, I would write nausea-. For example, I could now enter severe nausea. Now I could write here, the current value is, I think, a degree of severity. That’s how I would interpret it now. I would still be missing, I don’t know, from zero to ten, zero to four, that you just-. Because everyone evaluates it a little bit differently and if I now enter a five, because for me it’s from zero to ten, I don’t know how it is with my successor, how they would then enter it or evaluate it.”*

*(Nurse_11_#00:22:24#)*

The colour coding and presentation of symptoms in the PCM was not clear to all persons. For example, it was anticipated that each symptom would be assigned a specific colour and not that each colour would be assigned a particular intensity (e.g., pain red, shortness of breath blue). Here, the participants recommended that the colours should be labelled in such a way that it is directly comprehensible what exactly the colours mean (e.g., by a legend).

*“I first thought that red was for pain and shortness of breath was yellow, for example. That both are clicked.”*

*(Nurse_01_#00:06:34#)*

### 3.6. Catheter Management

A major problem with visualizing catheters was that those that had already been removed or pulled were visualized in the same dot symbol as catheters that were still on the patient. It was recommended that both statuses be clearly different to better assess the corresponding status. It was recommended that the symbol be changed to better assess the status.

*“Yes, exactly, the end times are a bit confusing, with the catheters. That everything, even if they continue, also has a start and an end point, so to speak. […] And if the others still continue, the line would actually just have to continue, and not also have, for me somehow, the same end point.”*

*(Nurse_01_#00:34:01#)*

### 3.7. Positioning

Documenting a patient’s current position was felt complicated and not intuitive. This was essentially related to the fact that only one state (e.g., left or right) could be documented for a specific time point (e.g., 7:00 a.m. patient is lying on the right side, 8:00 a.m. patient is lying on the left side) rather than for a period of time. Likewise, it should, however, be possible to be able to represent the clinical workflow as accurately as possible.

*“Yes, the thing with the times. […] So, I would rather enter a time span, really, from 10:00 to 10:30, instead of always clicking on sleep from ten and then again on awake, from, I don’t know, eleven. So I find that kind of, yeah, so-. I would find it easier if I could just type that in, really. Sleep, like I do with my marker, I draw the line and that’s it, yeah. But that would be so the only thing.”*

*(Nurse_01_#00:33:02#)*

Another request of the participants was that the current position of the patient be displayed directly in the plus-button menu.

### 3.8. Intake and Output

Observations regarding the intake and output component were essentially related to the general visualization and colour coding. In the PCM, documented information about the patient’s stool and urine was displayed in one line in different colours. For instance, information on urine is depicted in red, which was interpreted by some participants as “bloody urine”. Additionally, the use of the same symbol “circle” for documented information on stool and urine in the PCM and the complete masking of information when urine and stool were documented at the same time caused confusion.

In addition, it was perceived as confusing that stool and urine were displayed in the same circular form. When stool and urine were indicated at the same time during documentation, the display overlapped, and it was difficult to ensure that both entries were seen. It was requested that the documentation of stool and urine should be done in separate lines so that there is no overlap in the presentation.

*“So I would have said now, red means that the urine somehow, that there was blood in it. Here is the colour brown, ah okay. In and out stool. That’s what’s confusing me right now. It says urine, but when I go to it [via mouse over], it says bowel movement. […] Okay, that’s maybe a little bit confusing because you get a little bit mixed up. Exactly. I would maybe somehow separate urine and stool, so there’s another extra line like we have in the curves now. I think that would make it a bit clearer.”*

*(Nurse_08_#00:04:04#)*

An automated fluid balance function that takes into account the specifics of care for children (e.g., measuring the weight of the diaper) was particularly emphasized.

*“Especially now with the balance sheet, for example, that I don’t have to calculate it myself later, that the PC spits it out for me when I can then close the balance sheet or something. That the PC then tells me, “Okay. We have now calculated this. The patient has drunk so and so many millilitres in total or litres and has excreted so and so much.” And it is still important that especially with our children who are diapered-. If, for example, there is stool in the Pampers and I weigh them, then I would document the number of grams of the Pampers as I have weighed them, plus loss or plus stool. Because that doesn’t actually count in the balance sheet, but I can’t separate it.”*

*(Nurse_05_#00:41:45#)*

### 3.9. Events

A few sole observations to the events component could be coded. Currently, the area in which event (e.g., drop in oxygen, crisis) saturation is documented is visualized at the bottom end of the PCM. The participants found it problematic that documented events were visualized at the lower end. This requires additional scrolling to the end in the PCM because it might be necessary to relate such information directly to vital signs.

In general, it should also be possible to specify the duration of an event and not just its mere occurrence.

*“If the patient now has an acute crisis, so to speak, so let’s say here would be because of me the saturation on nothing and then you would always have to scroll down to see [documented events]. Whether it can then be useful if the events slide upwards. So because if, so events are probably rather crises. […]. Because then I would rather want to know in the morning, I come somehow and think oh God, what was there. Then I don’t want to know whether he [the patient] was asleep or not, but what was there. But otherwise, I find it first of all somehow totally clear and easy to use and helpful. And I think that makes a lot of things easier, because you can see how long things have been there and need to be changed and so on.”*

*(Physician_15_#00:49:10#)*

### 3.10. Facilitating Conditions

Several statements indicate how a PCM in an EMR might be implemented and what is perceived to be useful in clinical practice. The process of documentation was perceived to be less time-consuming and more useful if the computer for documentation was placed directly near the patient’s bed. In addition, it is necessary to provide a sufficient number of places with computers for documentation. Moreover, it was considered important to provide sufficiently large screens.

*“So, in the end, you have the values practically on a piece of paper and then you just enter them, right? So that’s how I imagine it now. Or I have directly, have such a laptop at the bed, where I can carry it then directly in. Yes. Somehow. Otherwise, one writes oneself also again doubly, if I write it first on a scratch note I say times and then into the curve. If I were with the PC at the bed or such a laptop, then one could enter it directly. So, then I think it’s not so time-consuming now.”*

*(Nurse_04_#00: 14:17#)*

*“I still see the problem that actually everyone must have such a thing [e.g., computer, tablet], that you have to have space to sit down, I need a display which is good and clear. Where I also see difficulties is when I have such a small display and such a curve that it becomes confusing. […] You always like to look at statistics, whether the statistics are also such a small picture afterwards, but I don’t need that at all. If I always have such a small display afterwards, that doesn’t help me either. So, I think the hardware is actually important. And also the supply in the hardware.”*

*(Nurse_03_#01:10:33#)*

Concerns were expressed that electronic documentation may be time-consuming and problematic, especially for older users with lower digital literacy levels. In connection with this statement, concerns were expressed that older users in particular may have greater problems than younger users.

*“Well, I think the younger ones can cope with it better than the older ones. I count myself as one of the younger ones. We more or less grew up with this kind of thing. We do a lot more with [computers] than our older colleagues. I think they’ll really have to get to grips with it. I think it will be more of a burden than a relief for them. I could imagine that.”*

*(Nurse_07_#00:50:49#)*

## 4. Discussion

The aim of the study was to evaluate the acceptance of a novel PCM in the early stage of development integrated in an EMR from the perspective of its potential users in PCC. Particularly in the dimension’s performance expectancy, effort expectancy and facilitating conditions, we were able to identify factors that are of relevance from the perspective of the participants in the context of PPC. Most of the participants perceived the PCM as intuitive and self-explanatory during its application. The presentation of the information was perceived as clear and comprehensible.

Nevertheless, it was verbalized that the amount of unfamiliar information presented in the PCM was initially overwhelming. This observation is well known in the use of EMRs and is explained in the literature with the phenomenon of cognitive load. Cognitive load is understood as the amount of mental load imposed on a person when solving a particular task and the associated required capacity of the human working memory [49]. In studies that also intend to evaluate electronic solutions as reported here and in the implementation of respective solutions in clinical practice, it is imperative to pay attention to this phenomenon, as cognitive load is related to the development of burnout and distress in EMRs [50]. However, in this study, after a certain period of use, the PCM was considered easier to use and became more familiar.

Moreover, we could identify factors that may contribute to the cognitive load due to unnecessary navigations and display fragmentation. Display fragmentation occurs when relevant information is separated in different parts of the EMR and requires unnecessary scrolling [25,51]. For example, unnecessary up- and down-scrolling to relate events and symptoms to each other may contribute to cognitive load because it requires additional cognitive working memory to synthesize information [25,51]. Additionally, we could identify unnecessary navigation steps that required many clicks per task, for example, in switching between different views of the PCM. One of the main strategies for usability improvement is to reduce the number of clicks [51].

Another major problem in the usability of EMRs is the fragmentation of tasks and switching between different views and can negatively influence the user’s workflow [28]. However, this problem was not observed or verbalized in our study. Reasons for this may be that only one module of the EMR was tested or that the tasks were formulated in such a way that the occurrence of fragmentation of tasks is unlikely. Similarly, the use of the PCM in a simulated test environment can contribute to this. This topic should be considered in more detail during and after future adaptions, especially when the PCM is tested in interaction with other modules. In general, the management of information overload is particularly important in PPC due to the complexity of patient conditions and long courses of treatment in PPC [52]. This is because information overload in the application of EHRs potentially leads to higher error rates and consequently negatively affects patient safety [53].

Several context-specific needs were suggested by the participants for improvement regarding performance expectancy, which may positively impact user acceptance in PPC. For example, as our results may indicate, the current representation of the sleep patterns in PCM was not sufficient for the setting of PPC to the participants. As sleep disorders are highly prevalent in the context of PPC, a view to assess the progression of changes over 24 h should be added as a further improvement of the PMC [54,55]. Additionally, regarding the documentation of symptoms, numerous participant requests were identified that could lead to an improvement of the documentation. In particular, it can be deduced from the users’ statements that there is a desire for clarity in the interpretation of the symptoms. Differentiated documentation is of high importance for the symptom management of children in palliative care settings [56]. Therefore, care should be taken in the development of EMR in PPC documentation of symptoms to ensure the specific requirements in PPC [57]. In this context, scales specific to the paediatric population must be used for the external assessment of pain, such as the paediatric pain questionnaire [58].

In addition, participants suggested other important improvements, such as an automatic fluid balance function or a more appropriate location and presentation of body weight in the PCM. Taking these functions into account in the further development of the PCM may also to be considered important in order to positively influence user acceptance.

Regarding facilitating conditions that contribute to acceptance, participants verbalized concerns regarding a sufficient infrastructure, such as appropriate sizes of computer screens or a sufficient number of computers. This corresponds to the results of a systematic review of Tsai et al. that identified limited access to or a small number of computers as an important barrier for the implementation of EMRs in general [17].

Additionally, the concerns that electronic documentation could be more time-consuming and problematic for older users with low digital literacy than paper-based documentation are not unfounded. A study in Finland identified that the age of the user and digital competences in particular are associated with the psychological distress of nurses who are using EMRs [28]. A qualitative study showed that nurses with low digital literacy skills may experience feelings of stress, frustration, incompetence, or postponing or avoiding the use of digital technologies in general [59]. Furthermore, continuous on-the-job training and collegial support by digitally experienced colleagues will be necessary [59]. However, a recent systematic review has shown that poor and insufficient training of users is considered a barrier to the use of EMRs, regardless of the age of the users [17].

The methodological approach using CTA must be taken into account when reviewing the results. As a recent survey showed, nurses and physicians are willing to participate in the development of health information technologies in clinical settings, and effective methods to obtain end-use feedback in clinical settings are underutilized [60]. By applying the CTA with the task-oriented approach, we were able to collect fine-grained information on tasks that nurses and physicians perform in their daily practice on the PPC unit [51,61]. This approach can be considered helpful in matching the functions of the PCM with the clinical workflow, which is an important factor of acceptance [62]. In addition, users were able to verbalize their wishes for improvements, which is seen as a key goal of participatory development [31]. Such information cannot be collected with quantitative methods, which are usually used retrospectively and summatively regarding the evaluation of technologies [61]. Moreover, it is not possible to make individual suggestions for improvement through such approaches to evaluation [42].

Furthermore, the theoretical approach based on UTAUT can also be considered useful and practicable. This approach has made it possible to systematize and formulate specific suggestions for improvement and redesign of the PCM. This is confirmed by studies in other contexts that have applied UTAUT in the context of participatory development of digital interventions [63]. It should be noted that in this study, we did not identify any statements related to social influence as a determinant of acceptance. It could be assumed that the test situation may have had an influence on this result, as it was very focused on the application of the PCM. In addition, a reason for this could be that the questions in our interview guide did not address this aspect specifically enough.

Building on the results of this study, it is necessary to first implement user requests and suggest revisions to establish the prerequisite necessary to ensure user acceptance. This is important because acceptance may affect both the quality of an application and its documentation, as well as user satisfaction [64]. Furthermore, it will be necessary to evaluate further modules (e.g., medication), as only one module could be evaluated in this study. Following this, further testing of the PCM should be performed.

For further evaluations, expert-based methods such as heuristic evaluation and cognitive walkthrough, which are also common methods in later development stages, could be used [51]. Another methodological approach would be the “near live clinical simulations” (NLCS), in which participants are exposed to the clinic with actors simulating real patients [65]. The use of such methodological approaches makes it possible to test EMRs under conditions that are more in line with real clinical situations. Especially in the field of PPC, which is characterized by patients with complex conditions and a multi-professional environment, such approaches would be beneficial due to vulnerable patients to prevent patient harm.

## 5. Limitations

The results have to be considered under different limitations. It can be assumed that the participants adapted their behaviour to the interview situation since two researchers were present as observers (Hawthorne effect) in a laboratory setting [66]. This is a well-known background for studies using the think aloud method in general, which must be considered when interpreting the results [65]. The open and explorative approach conducted here, often referred to as “relaxed-think aloud” or “interactive-think aloud”, is under discussion because it influences the behaviour of the participants during the interaction between the researcher and the participant [67]. Even though we have reduced the interaction with the participants to a minimum, it is possible that the interviewer influenced the behaviour [67]. Nevertheless, assuring a good balance between giving participants space to verbalize their thoughts on the one hand and asking questions and helping them, if necessary, seems to also be a good approach.

The entries that the participants were asked to make into the PCM occurred outside of real clinical conditions and did not represent real patient data. It must also be taken into account that only one module of an EMR was tested. Therefore, it is likely that different opinions and needs would emerge if the PCM was used under real clinical conditions, including other modules of the EMR, especially with regard to the experience of cognitive load [68].

Due to the sampling method, it is likely that people who had a particular interest in the application of the PCM took part in the study [66]. Since those who did not participate may have had reservations about EMRs or the chosen methodology, interesting insights into their views and desires could not be captured. Therefore, in future studies, it is important to find ways to recruit these persons to participate in such studies. For example, recruitment strategies should be more specifically adapted to the needs of such users, for instance, by removing fears of application in the study material or testing in a more familiar environment.

It must be emphasized that participants had not received any specific training in using the PCM. For that reason, it is not surprising that some participants initially perceived the flood of information in the PCM large. It can be assumed that both the application of the PCM and the quality of the documentation will increase if training sessions are carried out before implementation [69].

Finally, it must be mentioned that the results relate to the context of the German health care system. Therefore, the transfer of the results must be considered with caution.

## 6. Conclusions

The involvement of users in the development of EMR is important to meet the specific requirements in PPC and to ensure a high probability of user acceptance. This is relevant in this setting, as the patients are vulnerable to medical errors. Despite this, the PCM is considered intuitive and self-explanatory in the application from a user perspective. Special attention should be devoted to the management of information overload due to the amount of information on treatment in PPCs. A fine-grained approach, considering clinical tasks, is of particular importance. Further studies should be conducted under more realistic clinical conditions in PPC.

## Figures and Tables

**Table 1:** Participants’ characteristics.

Sex	n (%)
Female	11 (68.75)
Male	5 (31.25)
Age in years (SD)	42 (12)
Profession	
Physician	6 (56.25)
Nurse	10 (43.75)
Years of PPC experience	
0–9	6 (37.5)
10–20	5 (31.25)
>20	5 (31.25)
Years of experience in current position	
0–9	13 (81.25)
10–20	2 (12.5)
>20	1 (6.25)
Experience in professional use of EMR	6 (37.5)
Experience in professional use of EMR in years	
0–4	4 (66.67)
5–8	1 (16.76)
≥9	1 (16.67)

**Table 2:** Main categories and subcategories of acceptance factors.

(1) **General Observations**	
Intuitive application and familiarization Clearly arranged and concise overview Advantages and concerns of electronic documentation Transparency and traceability	
**(2)** **Performance Expectancies**	**(3)** **Effort Expectancies**
General view and plus-button	
Finer scaling	Unnecessary navigation action during switching views Change of view during retrospective entries Selection of dates retrospectively Differentiated presentation of symbols in the legend
Vital signs	
Location of body weight Depiction of body weight	Updating data of body weight Display of legend
Sleep patterns	
Detailed view of 24-h protocol	Documenting of time periods
Symptom observation	
Indicate the exact intensity (on a scale of 1–10) and add command function Captured sophisticated and standardized measures Text module for comment function More details on symptom documentation Flexible structure of symptom documentation Adding specific scales for the assessment of pain Labelling of newly appeared symptoms necessary	Display of legend Add colour coding in the legend of the symptoms related to the intensity of the symptoms
Catheter management	
Integration of laboratory results possible Add additional information for catheters	Clear visualizing of catheters status
Positioning	
Add additional features for the description regarding positioning Show the current position of the patient in the main menu of the plus-button Enable documentation of specific aids to support positioning	Documenting of time periods Show the current position as text in the timeline
Intake and output	
Add automated fluid balance function Add additional features for the description regarding output characteristics, (e.g., cloudy urine, yellow secretion)	Show stool and urine in separate lines Use different symbols for stool and urine
Events	
Visualized information on events related to vital signs Specify the duration of an event	
**(4)** **General Observations**	
Intuitive application and familiarization Clearly arranged and concise overview Advantages and concerns of electronic documentation Transparency and traceability	

## Data Availability

The corresponding datasets are available from the corresponding author upon reasonable request.

## Appendix A

**Elements of the Plus-Button Menu**

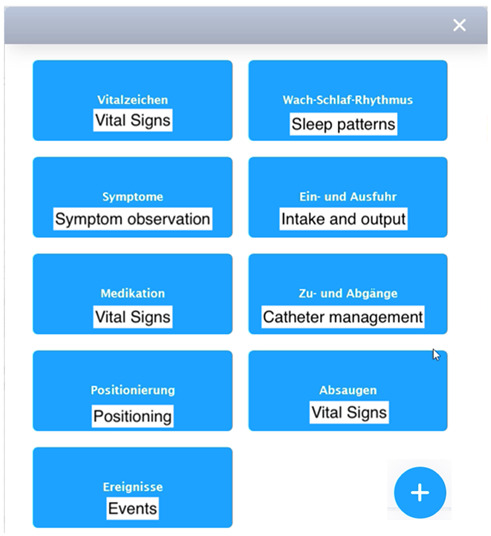

General view

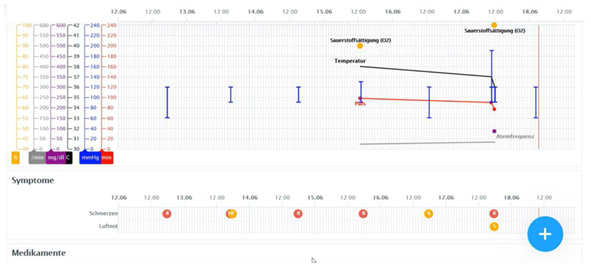

## Appendix B

**General View and Plus-Button**
- You can view the (vital signs) curve in different views, e.g., 3 days at a glance or 7 days at a glance. Look at the entries in the curve in the different views: 1 day, 3 days, 7 days - Find out how to click back or forward one day at a time - Look at the entries of the last weeks. Then, look at the day of the hospital admission. What was documented? - Click the blue button with the plus sign (+) at the bottom right of the screen to obtain an overview. This is called the “plus-button”
Vital signs
- Use the plus-button (+) at the bottom right of the screen to document blood pressure, pulse and saturation of your choice and save - Document a body weight of the patient and save. See if the weight is displayed in the header at the top. - Document a blood pressure and pulse taken 3 days earlier - Look at what values were entered for blood pressure and pulse on 15 May 2020 and designate the values.
Sleep patterns
- Document that the patient slept from 01:00 to 06:00 tonight - Document that the patient was awake from 06:00 to 10:00 this morning - Document that the patient slept from 10:00 am to 12:00 pm this morning - Check that everything is displayed correctly in the curve view
Symptom observation
- Document three symptoms with any comment of choice and save - Make sure that the comments on the symptoms have been copied correctly - Look if symptoms have already been documented in the last week times
Catheter management
- Name how many catheters are placed to the patient - Name when the catheters were placed - Document a new catheter of your choice and save - Document that you removed one of the catheter
Positioning
- Document that the patient was positioned on the left side (30 degrees) from 08:00 to 10:00 this morning - Document that the patient was on the right side (30 degrees) from 10:00 am to 12:00 pm this morning - Document that the patient sat on the edge of the bed from 12:00 a.m. to 12:30 p.m. this morning
Intake and output
- Document an output of 200 mL of urine - Document stool from this morning with any description - Verify that their documentation is visible in the chart view
Events
- Document an event from 08:00 this morning with any comment - Look at the comment in the curve view and check if everything was transferred correctly - Delete the entire entry and check if everything has been deleted correctly

## Appendix C

**Interview Guide**
- What were your first thoughts when you started working with the PCM? - What did you like and what did you not like? - Do you have the impression that it was easy for you to use? - Do you think the PCM helps you to get a better overview of the patients? - What do you think about how your colleagues use the PCM? - Do you have any other comments or wishes regarding the PCM?
